# 6q25.1-q25.3 Microdeletion in a Chinese Girl

**DOI:** 10.4274/jcrpe.galenos.2020.2020.0008

**Published:** 2021-02-26

**Authors:** Mian-Ling Zhong, Ye-Mei Song, Chao-Chun Zou

**Affiliations:** 1 Children’s Hospital of Zhejiang University Faculty of Medicine, Department of Endocrinology; National Clinical Research Center for Child Health, Huzhou, China; 2 Huzhou Center Hospital, Clinic of Pediatrics, Huzhou, China

**Keywords:** 6q25 microdeletion, facial dysmorphism, growth retardation, intellectual disability, language delay

## Abstract

Deletions of the long arm of chromosome 6 are rare and are characterized by great clinical variability according to the deletion breakpoint. Herein, we reported a 3-year-old girl evaluated for facial dysmorphism (long and connected eyebrows, big mouth, wide nasal bridge, high palatine arch, low set ears, and thin hair), growth retardation, intellectual disability, and language delay. Chromosomal microarray analysis revealed an 8.1-Mb deletion within 6q25.1-q25.3 ([hg19] chr6: 152,307,705-160,422,834) comprising 31 genes. Dysmorphic features, microcephaly, intellectual disability, language delay, growth retardation, and corpus callosum dysgenesis were commonly reported. Hence, 6q25 microdeletion is a rare condition. In patients with dysmorphic features, microcephaly, growth retardation, intellectual disability, language delay and corpus callosum dysgenesis, 6q25 microdeletion should be considered in the differential diagnosis and chromosomal microarray analysis should be performed to confirm the diagnosis.

**What is already known on this topic?**

6q25 microdeletion, a rare chromosome disorder, has been associated with growth restrictions, abnormal head shape, craniofacial anomalies, hypotonia, seizures, and mild to moderate intellectual disability.

**What this study adds?**

We reported a Chinese patient with an 8.1-Mb deletion involving 6q25.1-q25.3. Our patient shared the phenotypic features of the 6q25 microdeletion, including dysmorphic features with dysgenesis of the corpus callosum, growth retardation, intellectual disability, and language delay. In patients with dysmorphic features, microcephaly, growth retardation, intellectual disability, language delay and corpus callosum dysgenesis, 6q25 microdeletion should be considered in the differential diagnosis and chromosomal microarray analysis should be performed to confirm the diagnosis.

## Introduction

6q25 microdeletion, a rare chromosome disorder, has been associated with growth restrictions, abnormal head shape, craniofacial anomalies, hypotonia, seizures, and mild to moderate intellectual disability (1,2). Although a genotype–phenotype correlation has initially been proposed based on the proximal, medial, and distal location of the deletions, the reported cases with molecular karyotyping showed significant clinical heterogeneity, even with overlapping deletions (3,4). Most cases also share delayed verbal communication abilities, although detailed descriptions of speech have not generally been reported (5). 6q25 microdeletion has been extremely rare since the first report in 1975 (6). Herein, we reported a 3-year-old girl with 6q25 microdeletion to highlight this rare condition.

## Case Report

The female patient was the first child of healthy non-consanguineous parents. Family history was negative for neurological disorders, behavioral problems, or congenital anomalies. She was born at 39^+4^ weeks gestation via vaginal delivery with uneventful pregnancy. Her birth weight and length were 2,600 g and 48 cm without documented occipital frontal circumference and APGAR. After birth, feeding difficulty was noted without history of nasal feeding. Her global development was delayed, with no improvement in her developmental skills with age. She could sit at the age of 15 months, and crawl at the age of two years old. She could not walk without support at the age of 37 months. She could only call “Dad” and “Grandma” at the age of 39 months.

She was first presented to our outpatient clinic at the age of 37 months because of growth retardation, intellectual disability, and language delay. Physical examination showed a height of 85.8 cm [<-2 standard deviation (SD)] and a weight of 11.8 kg (-1 ~ -2 SD). Characteristic facial dysmorphism, including long and connected eyebrows, big mouth, wide nasal bridge, high palatine arch, low set ears, and thin hair was noted. The heart, lung, abdomen, limb, and muscle tension were unremarked. Brain magnetic resonance imaging (MRI) revealed dysmorphism of the corpus callosum and stronger T2 signal at basal ganglia.

**Figure 1:**
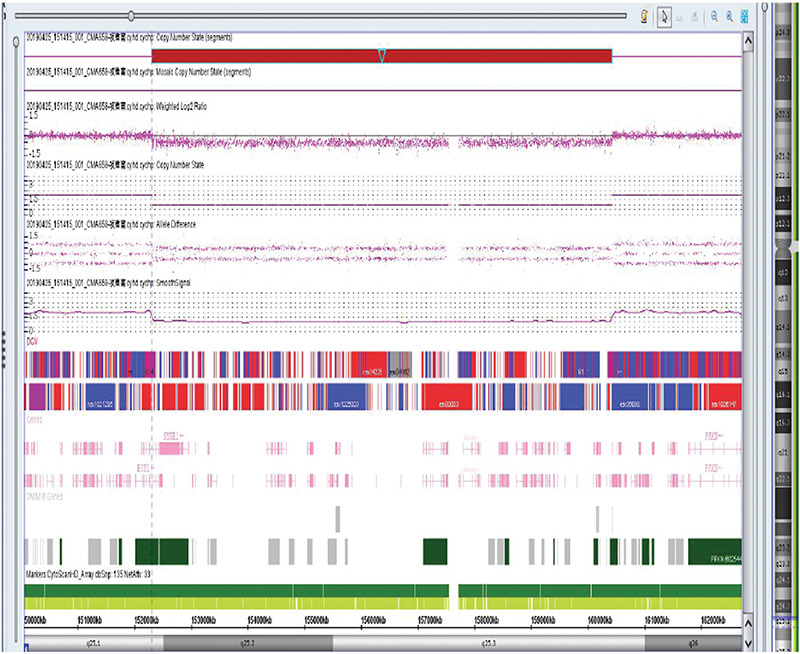
Chromosomal microarray analysis showed an 8.1-Mb deletion in 6q25.1-q25.3 (152,307,705-160,422,834), which covered 31 genes (100×65.9 mm)

Standard chromosome banding analysis performed in a local hospital reported “balanced translocation” in chromosome 4 and 8 for this patient and no abnormality for her parents. Chromosomal microarray analysis (CMA) (CMA, CytoScan® HD, Affymetrix) performed in our Medical Genetics Laboratory did not find any microdeletion or microduplication in chromosome 4 or 8, but identified an 8.1-Mb deletion in 6q25.1-q25.3 ([hg19]chr6:152,307,705-160,422,834), which covered 31 genes *(ESR1, SYNE1, MYCT1, VIP, FBXO5, MTRF1L, RGS17, OPRM1, CNKSR3, SCAF8, TIAM2, TFB1M, NOX3, ARID1B, SNX9, SYNJ2, SERAC1, GTF2H5, TMEM181, DYNLT1, EZR, RSPH3, TAGAP, FNDC1, SOD2, WTAP, ACAT2, TCP1, MRPL18, MAS1 *and *IGF2R*), as shown in Figure 1.

## Discussion

Interstitial deletions of the long arm of chromosome 6 are rare. Since the first report in 1975 (6), the number of patients that have been described in the medical literature remains few (1,2,5,7,8,9,10,11,12,13,14,15,16,17). The phenotype of this syndrome is variable and depends on the breakpoints, location and size of the deletion. Facial dysmorphism, hand malformations, heart defects, microcephaly, intellectual disability, epilepsy, and other neurodevelopmental and neuropsychiatric conditions have been reported. A comparison of the clinical characteristics of our patients with those reported in the literature is shown in Table 1. The clinical features observed in our patient and the other 22 previously reported patients showed that 22 (95.7%) had dysmorphic features, 21 (91.3%) had intellectual disability and language delay, 16 (69.6%) had microcephaly, 14 (60.9%) had growth retardation, and 10 (43.5%) had corpus callosum dysgenesis. The clinical characteristics of our patient overlap with several of these patients. The most common ones include growth retardation, intellectual disability, language delay, and dysmorphic features as well as dysgenesis of the corpus callosum, while microcephaly, hearing loss, limb anomalies and genital hypoplasia are not noted in our patient. These differences may be partly attributed to the varying size and breakpoints of the deletion and more importantly, the gene content of the deleted segment (18). In our patient, CMA showed an 8.1-Mb deletion of 6q25.1-q25.3, which covers 31 genes and only four of these genes (*ARID1B, IGF2R, TIAM2* and *SYNJ2*) have been associated with pathogenicity. Short stature was observed in our patient, while it was not noted in some previous cases (5,8,11,12,15). This may be due to the deletion of the *IGF2R* gene in our patient. Most other case reports do not specify the deleted genes so further comparison is not possible. However, studies of mice have supported a major role for the *IGF* receptor pathway in growth: knockout of *IGF1, IGF2*, or *IGF1R* results in growth retardation, whereas overexpression of *IGF2* results in overgrowth (19,20). The identification of an* IGF2* mutation in patients with growth restriction suggests that *IGF2* is not only a mediator of intrauterine development but also contributes to postnatal growth (21). The importance of other deleted genes and their contribution to the 6q25 microdeletion are uncertain at this time. Additionally, links between brain anomalies and language delay has been noted in the literature. For instance, de Vasconcelos Hage et al (22) reported that 13 cases of perisylvian polymicrogyria and three cases of corpus callosum hypoplasia were found in 17 patients with language impairment. In typically developing young children, the developmental rate of the splenium of the corpus callosum was associated with vocabulary size (23). In individuals with disfluent speech, the anterior corpus callosum showed significantly lower fractional anisotropy than that of typical controls (24). Hence, in patients with dysmorphic features, microcephaly, intellectual disability, language delay, and corpus callosum dysgenesis, 6q25 microdeletion should be considered in the differential diagnosis and CMA should be performed to confirm the diagnosis.

The mechanism of 6q25 microdeletion is still unclear. The smallest critical region described so far for 6q25 microdeletion have restricted to a 6q25.3 region including two protein-coding genes, *ARID1B *and *ZDHHC14* which was considered to be responsible for the cognitive impairment and brain anomalies observed in their patients (15). The core phenotypic characteristics associated with the 6q25 microdeletion have been observed in a child with a deletion involving only *ARID1B* which suggested that ARID1B may be one key gene associated with these features (16). Additionally, *ARID1B* has been associated with multiple syndromes characterized by developmental delay and intellectual disability, such as Coffin-Siris syndrome, and with non-syndromic intellectual disability. It is reported that *ARID1B *is of great importance for normal human brain development and function. In one study, the phenotype-genotype correlation in seven patients who had various-sized deletions including *ARID1B,* has shown that haplo-insufficiency of* ARID1B* is related with intellectual disability, speech impairment, and autism as well as corpus callosum abnormalities (25). Therefore, haploinsufficiency of *ARID1B *appears to be responsible for the clinical findings in our patient.

## Conclusion

In summary, 6q25 microdeletion is a rare condition. In patients with dysmorphic features, microcephaly, growth retardation, intellectual disability, language delay, and corpus callosum dysgenesis, 6q25 microdeletion should be considered in the differential diagnosis and CMA should be performed to confirm the diagnosis. MRI of the brain should be considered in all patients with deletions involving 6q25.

## Figures and Tables

**Table 1:**
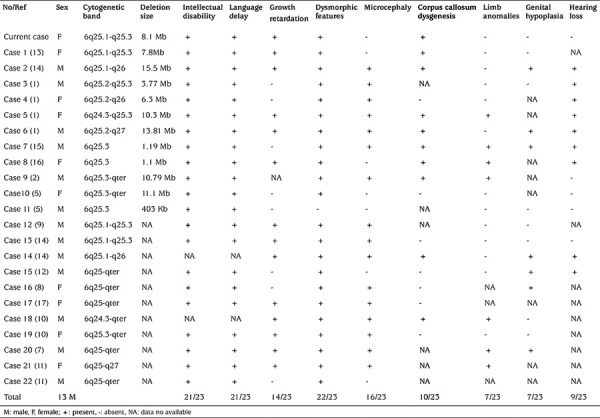
Clinical features observed in our patient and other reported patients with 6q25 microdeletion
